# Identification of miR-515-3p and its targets, vimentin and MMP3, as a key regulatory mechanism in esophageal cancer metastasis: functional and clinical significance

**DOI:** 10.1038/s41392-020-00275-8

**Published:** 2020-11-27

**Authors:** Hui-Fang Hu, Wen Wen Xu, Wei-Xia Zhang, Xin Yan, Yang-Jia Li, Bin Li, Qing-Yu He

**Affiliations:** 1 Key Laboratory of Functional Protein Research of Guangdong Higher Education Institutes and MOE Key Laboratory of Tumor Molecular Biology, Institute of Life and Health Engineering, Jinan University, Guangzhou, China; 2 Guangdong Provincial Key Laboratory of Bioengineering Medicine and MOE Key Laboratory of Tumor Molecular Biology, National Engineering Research Center of Genetic Medicine, Institute of Biomedicine, College of Life Science and Technology, Jinan University, Guangzhou, China

**Keywords:** Metastasis, Non-coding RNAs, Gastrointestinal cancer, Tumour biomarkers, Drug development

## Abstract

Metastasis is the main factor of treatment failure in cancer patients, but the underlying mechanism remains to be elucidated and effective new treatment strategies are urgently needed. This study aims to explore novel key metastasis-related microRNAs (miRNAs) in esophageal squamous cell carcinoma (ESCC). By comparing miRNA profiles of the highly metastatic ESCC cell sublines, we established through serial in vivo selection with the parental cells, we found that the expression level of miR-515-3p was lower in ESCC tumor tissues than adjacent normal tissues, further decreased in metastatic tumors, and moreover, markedly associated with advanced stage, metastasis and patient survival. The in vitro and in vivo assays suggested that miR-515-3p could increase the expression of the epithelial markers as well as decrease the expression of the mesenchymal markers, and more importantly, suppress invasion and metastasis of ESCC cells. Mechanistically, we revealed that miR-515-3p directly regulated vimentin and matrix metalloproteinase-3 (MMP3) expression by binding to the coding sequence and 3′untranslated region, respectively. In addition, the data from whole-genome methylation sequencing and methylation-specific PCR indicated that the CpG island within miR-515-3p promoter was markedly hypermethylated in ESCC cell lines and ESCC tumor tissues, which may lead to deregulation of miR-515-3p expression in ESCC. Furthermore, our preclinical experiment provides solid evidence that systemic delivery of miR-515-3p oligonucleotide obviously suppressed the metastasis of ESCC cells in nude mice. Taken together, this study demonstrates that miR-515-3p suppresses tumor metastasis and thus represents a promising prognostic biomarker and therapeutic strategy in ESCC.

## Introduction

Tumor metastasis is one of the most important hallmarks of cancer that leads to poor prognosis of cancer patients. Epithelial–mesenchymal transition (EMT), a crucial process by which polarity and cell attachment decrease accompanied with the downregulated expressions of epithelial molecules and the upregulated expressions of mesenchymal molecules. Vimentin is one of the most commonly used markers for EMT, and a large number of studies have reported that vimentin is closely related to cancer metastasis.^1–5^ In addition, matrix metalloproteinases (MMPs), such as MMP-1, 2, 3, 6, 9, and other members, are considered as the major proteolytic enzymes that hydrolyze matrix components and destroy tissue barriers to promote cancer metastasis. MMP2 can degrade the extracellular matrix such as type IV collagen fiber, increase the invasion and distant metastasis,^6^ whereas MMP9 can destroy the extracellular matrix including type IV, V collagen, and gelatin.^7^ Among the family members, MMP3 is special because it not only degrades the extracellular matrix and basement membrane,^8,9^ but also activates a variety of matrix metalloenzyme precursors, providing conditions for tissue invasion and metastasis during tumor progression. However, the underlying regulatory mechanism of the key drivers in invasion and metastasis is largely unknown.

MicroRNAs (miRNAs) are highly conserved, non-coding RNA products of ~22 nucleotides, which exist in a single-stranded form. miRNAs can lead to degradation of the specific target genes by binding with sequences in the coding sequence (CDS) or 3′untranslated regions (3′-UTRs) of target mRNAs,^10,11^ thereby inhibiting gene expressions.^12^ Increasing studies have shown that miRNAs have a significant role in regulating malignant phenotypes including cell proliferation, differentiation, apoptosis, and metastasis,^13–16^ and have the potential as effective diagnostic and prognostic markers in cancer. However, the functional and clinical significance of miRNAs in metastasis of esophageal squamous cell carcinoma (ESCC) remains to be explored.

In our study, by comparing miRNA profiles of the highly invasive and metastatic ESCC cell sublines, which we established through serial in vitro and in vivo selections, with the parental cells, several miRNAs most differentially expressed in the highly metastatic ESCC cells became our research focus. Among the candidate miRNAs, miR-515-3p expression was confirmed to be downregulated in the highly invasive and metastatic sublines and correlated with metastasis status and prognosis of the cancer patients. However, up to now, the biological function of miR-515-3p in cancer metastasis and its upstream regulatory mechanism has not been reported. Moreover, the role of miR-515-3p in ESCC and clinical significance is unknown. Here, a series of gain- and loss-of-function experiments and in vivo and in vitro functional experiments were performed to examine the effect of miR-515-3p on cancer invasion and metastasis as well as the potential upstream and downstream mechanisms. The therapeutic potential of systemically delivered miR-515-3p oligonucleotide in the treatment of cancer metastasis was examined.

## Results

### Deregulation of miR-515-3p is correlated with patient survival in esophageal cancer

**Fig. 1:**
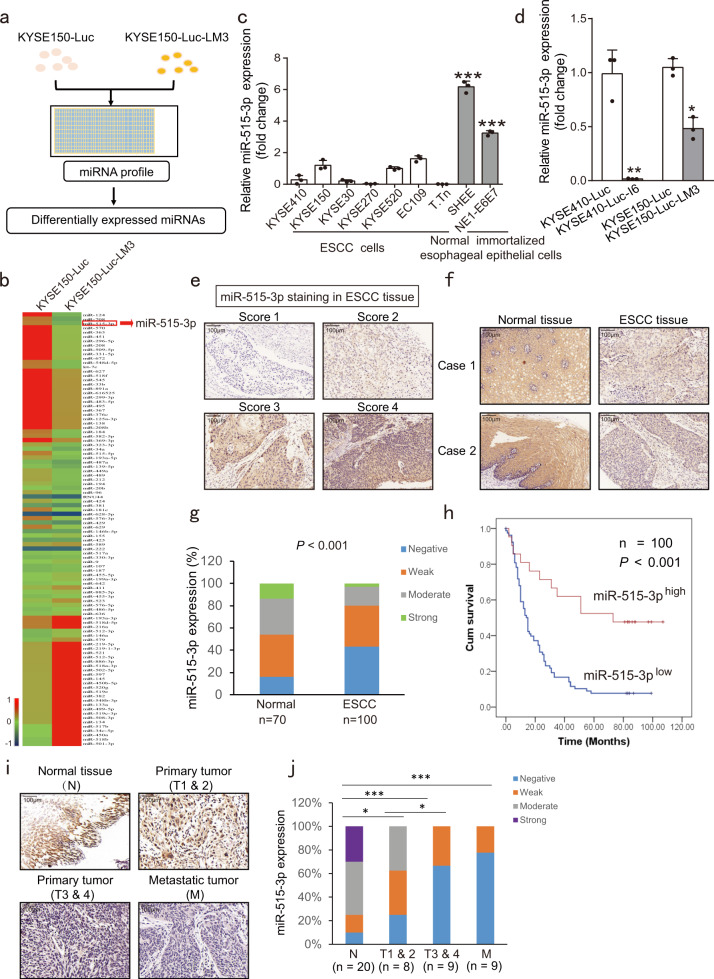
Identification of miR-515-3p as a potential regulator of tumor metastasis and its clinical significance in ESCC. **a** Experimental diagram of miRNA profiling to find the expressed differentially miRNAs in KYSE150-Luc-LM3 and parental cells. **b** Heatmap of the miRNA profiles of KYSE150-Luc-LM3 and KYSE150-Luc cells. Red color for high abundance and blue color for low abundance. **c** Comparison of the expression of miR-515-3p in ESCC cells and the immortalized normal esophageal epithelial cells by qRT-PCR. **d** Comparison of miR-515-3p expression in KYSE150-Luc-LM3 and KYSE410-Luc-I6 cells we established, and the parental cells by qRT-PCR. **e** Representative results of different staining scores for miR-515-3p in ESCC. **f** Representative results of in situ hybridization staining for miR-515-3p expression level in paired ESCC tissues and normal tissues. **g** Comparison of miR-515-3p expression in 100 cases of ESCC primary tumor tissues and 70 cases of adjacent normal tissues. **h** Based on miR-515-3p expression, the overall survival of 100 patients with ESCC was analyzed by Kaplan–Meier plot. **i**, **j** The miR-515-3p expression in 46 cases of normal tissues, primary tumors (T1 & 2, T3 & 4) and metastatic tumors were detected by in situ hybridization

To screen and identify which miRNAs regulate cancer metastasis, the miRNA profiles of highly metastatic ESCC subline KYSE150-Luc-LM3 (Supplementary Fig. S1a) obtained through serial in vivo selection and the KYSE150-Luc parental cells were compared (Fig. 1a, b). Expression levels of some well-known metastasis-suppressive miRNAs such as miR-124^17^ and miR-708,^18^ were markedly decreased in the highly metastatic ESCC subline, strongly suggesting that this model is useful in profiling metastasis-associated miRNAs. Among the top 50 downregulated miRNAs in KYSE150-Luc-LM3 cells, 6 miRNAs, which have not been previously reported to be involved in cancer metastasis were selected in a pilot study to examine their effects on cancer cell invasion. In this study, the results showed that miR-515-3p overexpression obviously repressed the invasive potential of KYSE150-Luc-LM3 cells (Supplementary Fig. S2a, b), which became our research focus. And miR-515-3p expression was lower in ESCC cells as compared with the normal immortalized esophageal epithelial cells (Fig. 1c). We also established the highly invasive sublines KYSE410-Luc-I6 (Supplementary Fig. S1b) through serial in vitro selection,^19^ and found that miR-515-3p expression further decreased in the highly invasive and metastatic sublines KYSE410-Luc-I6 and KYSE150-Luc-LM3 (Fig. 1d).

To assess the clinical significance of miR-515-3p in ESCC, miR-515-3p expression was analyzed by in situ hybridization in a tissue microarray containing 100 cases of primary ESCC tumor tissues and 70 cases of adjacent normal tissues (Fig. 1e). The majority of the tumor tissues had weaker miR-515-3p staining than corresponding normal tissues (Fig. 1f). As indicated in Fig. 1g, 45.7% (32/100) of normal tissues showed high miR-515-3p expression, whereas only 22.0% (22/100) of tumor tissues showed high miR-515-3p expression. Low miR-515-3p expression was significantly correlated with advanced stage and lymph node metastasis in cancer patients (Table 1). More importantly, patients with low expression of miR-515-3p had obviously shorter survival (median survival = 22.4 months) than the patients with high miR-515-3p expression (median survival = 57.0 months) (Fig. 1h).

**Table 1:** Correlation between miR-515-3p expression levels and clinicopathological parameters in 100 cases of esophageal cancer

Variable	*n*	Low miR-515-3p	High miR-515-3p	*P*-value
Age (years)				
≤55	19	17	2	
>55	81	62	19	0.212
Gender				
Female	26	20	6	
Male	74	59	15	0.762
T-stage				
1/2	16	10	6	
3/4	84	69	15	0.077
N-stage				
N0	45	30	15	
N1	55	49	6	0.006
M-stage				
M0	100	79	21	
M1	0	0	0	1.00
Grade				
I & II	73	54	19	
III & IV	27	25	2	0.042
Stage				
1 & 2	48	32	16	
3 & 4	52	47	5	0.003

In addition, the correlation of miR-515-3p expression and metastasis was analyzed using a TMA of ESCC containing 46 cases of normal tissues, primary tumors (T1 & 2, T3 & 4) and metastatic tumors. As showed in Fig. 1i, j, the majority of the normal tissues had higher miR-515-3p expression than primary tumors (T1 & 2, T3 & 4) and metastatic tumors, and the expression of miR-515-3p was associated with the tumor stage and metastasis. Analysis of data from the OncomiR Cancer Database^20^ indicated that miR-515-3p expression was markedly correlated with the survival of the patients with esophageal cancer (Supplementary Fig. S2c). Moreover, miR-515-3p expression was also associated with clinical parameters and survival in several other cancer types such as kidney renal papillary cell carcinoma (Supplementary Tables 1 and 2). These data indicated that miR-515-3p may be a novel biomarker for cancer diagnosis and prognosis.

### miR-515-3p is silenced in ESCC cell lines and tissues by promoter hypermethylation

**Fig. 2:**
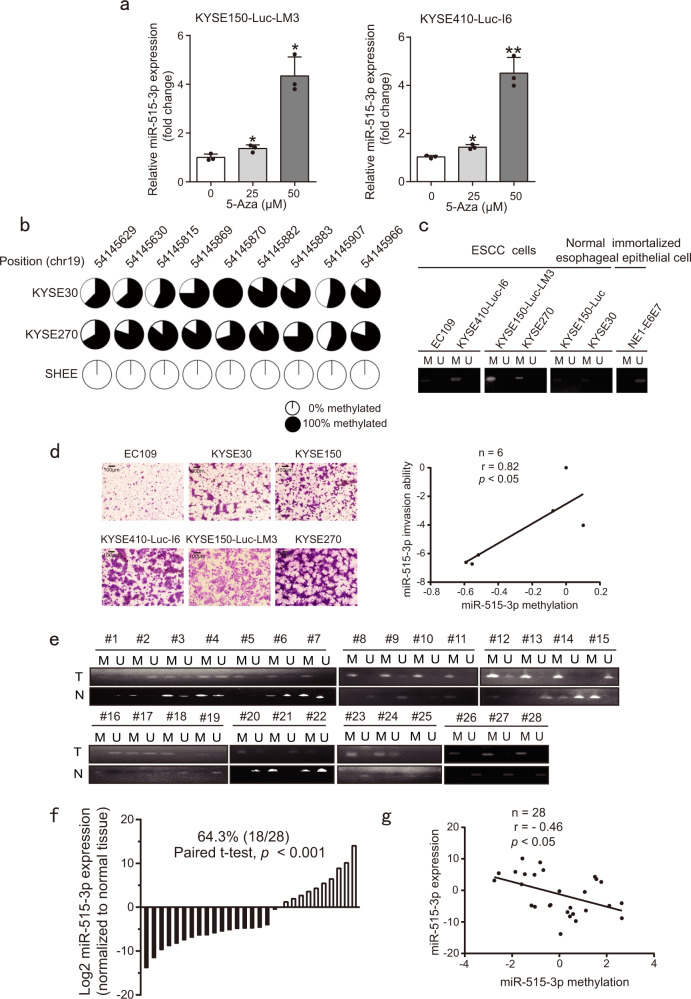
Promoter hypermethylation of miR-515-3p in ESCC cell lines and tissues. **a** miR-515-3p expression in cells treated with the indicated concentrations of 5-Aza was analyzed by qRT-PCR. **b** Whole-genome methylation sequencing was used to analyze DNA methylation status of promoter region of miR-515-3p. Each plot showing the methylation percentage of each site. **c** The hypermethylation of miR-515-3p in ESCC cells was detected by methylation-specific PCR. **d** Comparison of the invasive potential of ESCC cell lines and the correlation with methylation level of miR-515-3p was analyzed. **e**, **f** The hypermethylation and expression of miR-515-3p in 28 pairs of ESCC tissues and normal tissues was detected by methylation-specific PCR and qRT-PCR. **g** miR-515-3p expression was negatively associated with methylation level in ESCC tissues

In order to investigate whether downregulated miR-515-3p expression in ESCC is due to promoter hypermethylation, 5-Aza-2′-deoxycytidine (5-Aza), an epigenetic modifier results in DNA demethylation, was used to treat ESCC cells, and miR-515-3p expression was compared with control cells. We found that 5-Aza treatment markedly increased miR-515-3p expression in a dose-dependent manner (Fig. 2a). Whole-genome methylation sequencing was conducted to determine DNA methylation status in 2 ESCC cell lines and 1 immortalized normal esophageal epithelial cell line, and heavy methylation of the 5′CpG island was observed in the promoter region of miR-515-3p (Fig. 2b). Notably, the results from methylation-specific PCR revealed that the CpG island within miR-515-3p was significantly hypermethylated in ESCC cell lines examined, but not in immortalized normal esophageal epithelial cells (Fig. 2c). As shown in Fig. 2d, the invasion ability of ESCC cell lines was compared and the results showed methylation level of miR-515-3p was markedly positively correlated with the invasive potential of ESCC cell lines (*r* = 0.82, *p* < 0.05). We also determined the methylation level and expression level of miR-515-3p in 28 pairs of ESCC tumor and normal tissues (Fig. 2e, f). miR-515-3p was found significantly hypermethylated in 60.7% (17/28) of the ESCC tissues compared with their paired normal tissues (Fig. 2e), and 64.3% (18/28) of the ESCC tumor tissues had lower miRNA-515-3p expression than adjacent normal tissues (Fig. 2f). We found that miR-515-3p expression was negatively associated with its methylation level in 28 pairs of ESCC tissues and normal tissues (*r* = − 0.46, *p* < 0.05) (Fig. 2g). These data implied that promoter hypermethylation leads to the deregulation of miR-515-3p expression in ESCC.

### miR-515-3p inhibits ESCC invasion and metastasis

**Fig. 3:**
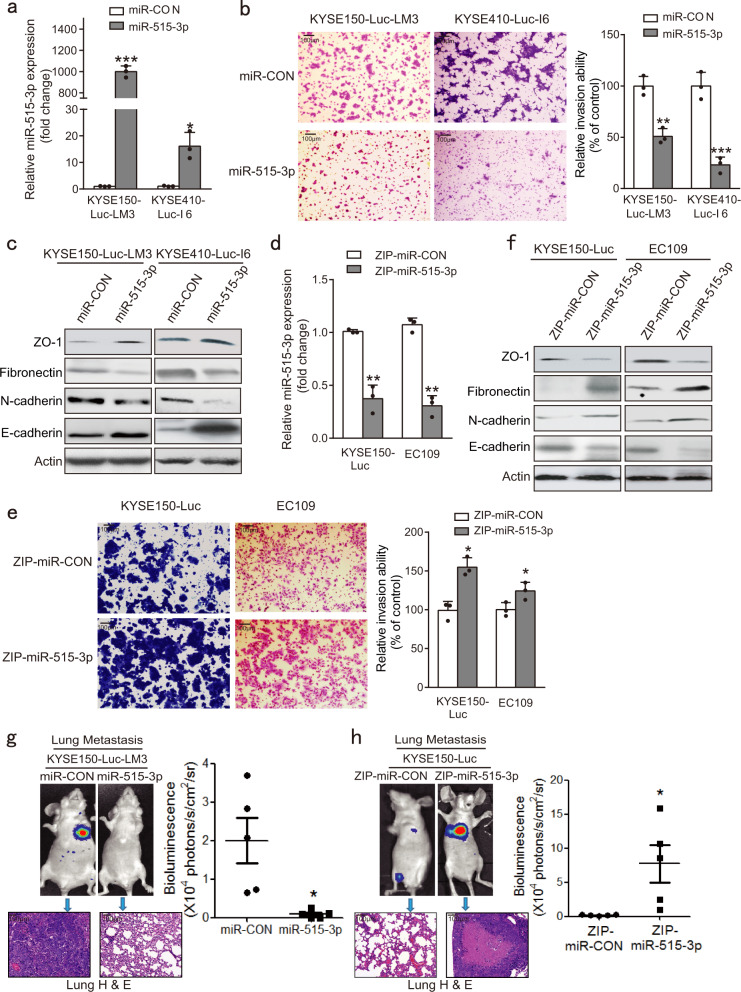
miR-515-3p inhibits ESCC invasion and metastasis. **a** miR-515-3p-overexpressing stable cell lines were established. **b** The invasive potential of miR-515-3p-overexpressing cells and control cells was performed by Chamber invasion assay. **c** ZO-1, fibronectin, N-cadherin, and E-cadherin expression levels in miR-515-3p-overexpressing cells and control cells. **d** miR-515-3p-knockdown stable cell lines were established. **e** Comparison of invasive potential of miR-515-3p-knockdown cells and control cells by chamber invasion assay. **f** ZO-1, fibronectin, N-cadherin, and E-cadherin expression in miR-515-3p-knockdown cells and control cells were analyzed by western blotting. **g** Left upper panel, lung metastasis in nude mice intravenously injected with KYSE150-Luc-LM3-miR-515-3p or KYSE150-Luc-LM3-miR-CON was detected by bioluminescence imaging. Left lower panel, histological evaluation (H & E staining) of lung metastasis derived from KYSE150-Luc-LM3-miR-515-3p or KYSE150-Luc-LM3-miR-CON cells. Right panel, quantification showing the effect of miR-515-3p overexpression on lung metastasis of ESCC cells. **h** Comparison of lung metastasis in the mice intravenously injected with KYSE150-Luc-ZIP-miR-515-3p and KYSE150-Luc-ZIP-miR-CON cells by bioluminescence imaging and H & E staining

The function of miR-515-3p in ESCC invasion and metastasis has not been reported previously. miR-515-3p-overexpressing stable cell lines were generated in the highly invasive and metastatic ESCC cells, KYSE410-Luc-I6 and KYSE150-Luc-LM3, which we established previously (Fig. 3a). The results showed that KYSE410-Luc-I6-miR-515-3p and KYSE150-Luc-LM3-miR-515-3p cells had lower invasive, migration and adhesion ability, as compared with corresponding parental cell lines expressing scrambled miRNAs (Fig. 3b and Supplementary Fig. S3a). EMT marker expression were analyzed, decreased fibronectin and N-cadherin expression as well as increased E-cadherin and ZO-1 expression were observed in miR-515-3p-overexpressing ESCC cells (Fig. 3c). On the other hand, we also successfully constructed miR-515-3p-knockdown ESCC cell lines, designated as KYSE150-Luc-ZIP-miR-515-3p and EC109-ZIP-miR-515-3p (Fig. 3d). As shown in Fig. 3e and Supplementary Fig. S3b, inhibition of miR-515-3p expression increased the invasive, migration, and adhesion ability of ESCC cells. We also found decreased E-cadherin and ZO-1 as well as increased fibronectin and N-cadherin expression in miR-515-3p-knockdown ESCC cells, as compared with their control cells respectively (Fig. 3f). Moreover, our results showed that miR-515-3p did not exert any change in proliferation and apoptosis of ESCC cells within 24 h (Supplementary Fig. S4a, b), eliminating the possibility that weaker invasion ability of miR-515-3p-overexpressing cells was due to the effect on proliferation and apoptosis.

Furthermore, for experimental metastasis assay, KYSE150-luc-LM3-miR-515-3p cells were intravenously injected into the tail vein of mice, and lung metastasis was monitored using bioluminescence imaging. The results showed that miR-515-3p significantly suppressed lung metastasis, which was confirmed by hematoxylin and eosin (H & E) staining of lung sections (Fig. 3g). In contrast, loss-of-function experiment showed that knockdown of miR-515-3p significantly promoted lung metastasis (Fig. 3h). Collectively, these results indicated for the first time that miR-515-3p may be a novel suppressor of cancer cell invasion and metastasis.

### miR-515-3p directly targets and inhibits vimentin and MMP3 expression

**Fig. 4:**
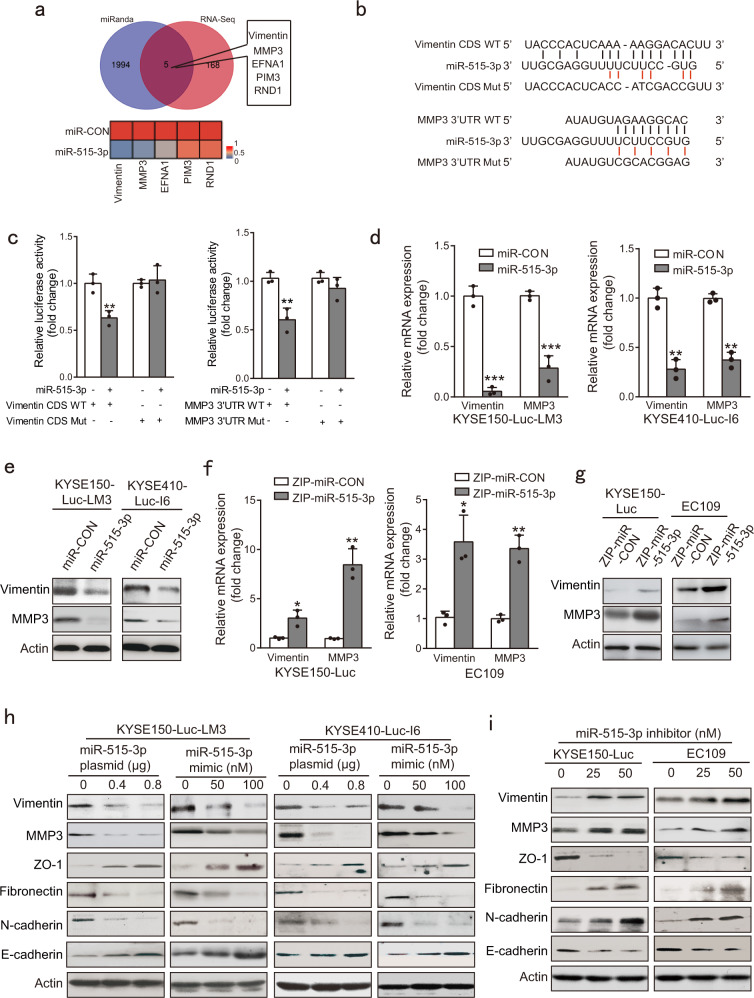
miR-515-3p directly targets vimentin and MMP3 to inhibit their expression. **a** miRNA-target prediction algorithm and RNA-seq were used to predict possible targets of miR-515-3p. **b** Alignment of miR-515-3p and its corresponding complementary binding sequence in vimentin CDS and MMP3 3′-UTR by miRanda bioinformatics algorithm (red indicates mutant site). **c** The miR-515-3p mimic was co-transfected with wild-type or mutant vimentin CDS (left panel) or MMP3 3′-UTR (right panel) in KYSE150 cells, and luciferase activity was determined. The Renilla luciferase-expressing vector pRL-TK was regarded as an internal control. **d**, **e** Vimentin and MMP3 expression in miR-515-3p overexpression cells and control cells were analyzed. **f**, **g** Comparison of vimentin and MMP3 expression in miR-515-3p-knockdown cells and control cells by using western blotting and qRT-PCR. **h** Vimentin, MMP3, and EMT markers expression in the ESCC cells transfected with miR-515-3p mimic or miR-515-3p-expressing plasmid as compared with control. **i** The miR-515-3p inhibitor increased expression levels of vimentin, MMP3, fibronectin, and N-cadherin, whereas reduced ZO-1 and E-cadherin expression in ESCC cells

To explore the molecular mechanism how miR-515-3p acts its biological function, gene profiles of KYSE150-Luc-LM3-miR-515-3p and KYSE150-Luc-LM3-miR-CON cells were compared by RNA-sequencing (Supplementary Fig. S5a), and 173 transcripts were found to be downregulated in KYSE150-Luc-LM3-miR-515-3p cells (fold change > 1.5) (Supplementary Fig. S5b). Simultaneously, miRNA-target prediction algorithm was used to recognize potential targets of miR-515-3p. With stringent criteria, a Venn diagram revealed that five genes were identified in both the methods as a potential target of miR-515-3p (Fig. 4a). Among the five overlapped genes, vimentin and MMP3, which have been widely recognized as key regulators in promoting cancer invasion and metastasis, drew our great attention to postulate that miR-515-3p may exert its suppressive effect on cancer mobility through its regulation on vimentin and MMP3.

Computational analysis suggested the possible direct binding sites for miR-515-3p to be in the coding sequence (CDS) of vimentin and 3′-UTR of MMP3, respectively (Fig. 4b). For verification, using 3′-UTR luciferase reporter assays, we found that transfection of miR-515-3p mimic in KYSE150 cells led to an obvious decrease in the relative luciferase activity of vimentin CDS and MMP3 3′-UTR, but there was no inhibition of luciferase activity when miR-515-3p mimic was co-transfected with mutant MMP3 3′-UTR or vimentin CDS (Fig. 4c). Significant decreases in vimentin and MMP3 expression at mRNA and protein levels were observed in the highly invasive and metastatic cell lines with stable overexpression of miR-515-3p (Fig. 4d, e). Conversely, expression levels of vimentin and MMP3 were upregulated in the ESCC cells with stable knockdown of miR-515-3p (Fig. 4f, g).

Moreover, gain- and loss-of-function experiments and western blotting analysis revealed that transient transfection of miR-515-3p mimic or miR-515-3p-expressing plasmid not only markedly reduced vimentin and MMP3 expression at mRNA and protein levels, but also decreased fibronectin and N-cadherin expression in ESCC cell lines (Fig. 4h and Supplementary Fig. S6a, b), whereas silencing of miR-515-3p with inhibitor had opposite effect on vimentin, MMP3 and EMT markers (Fig. 4i and Supplementary Fig. S6c).

These data make it obvious that miR-515-3p affects the expression levels of vimentin and MMP3 by binding to CDS and 3′-UTR region, respectively, thus suggesting that downregulation of miR-515-3p may lead to vimentin- and MMP3-mediated cancer invasion and metastasis.

### Vimentin and MMP3 mediate the effect of miR-515-3p on cancer cell mobility

**Fig. 5:**
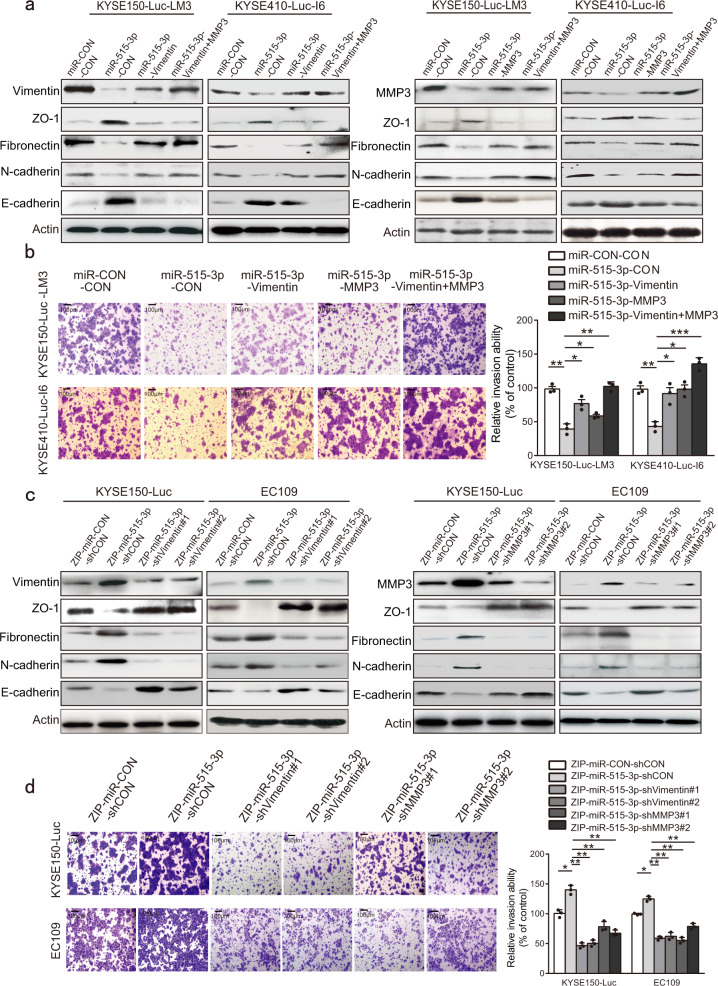
Vimentin and MMP3 mediate the effect of miR-515-3p on cancer cell mobility. **a** The I6 and LM3 cells co-overexpressing miR-515-3p and vimentin, or miR-515-3p and MMP3, were assayed for EMT marker expression by western blotting. **b** Comparison of the invasive potential of the cells co-overexpressing miR-515-3p and vimentin, or miR-515-3p and MMP3, as well as the control cells, by chamber invasion assay. **c**, **d** Expression of vimentin or MMP3 was further inhibited in the miR-515-3p-knockdown ESCC cells, and EMT marker expression and invasive ability of the cells were compared with control cells by western blotting (**c**) and chamber invasion assay (**d**), respectively

We next examined the importance of vimentin and MMP3 in the functional role of miR-515-3p in cancer metastasis. The I6 and LM3 cells co-overexpressing miR-515-3p and vimentin, or miR-515-3p and MMP3, or miR-515-3p, vimentin and MMP3 (KYSE150-Luc-LM3-miR-515-3p-vimentin, KYSE150-Luc-LM3-miR-515-3p-MMP3, KYSE150-Luc-LM3-miR-515-3p-vimentin+MMP3, KYSE410-Luc-I6-miR-515-3p-vimentin, KYSE410-Luc-I6-miR-515-3p-MMP3, KYSE410-Luc-I6-miR-515-3p-vimentin+MMP3), or miR-515-3p alone (KYSE150-Luc-LM3-miR-515-3p-CON, KYSE410-Luc-I6-miR-515-3p-CON), and the control cells (KYSE150-Luc-LM3-miR-CON-CON, KYSE410-Luc-I6-miR-CON-CON) were compared for the expression levels of EMT markers and their invasive and migration ability. Decreased fibronectin and N-cadherin expression and increased E-cadherin and ZO-1 expression were observed in the miR-515-3p-overexpressing cells, and these effects were abolished by ectopic expression of vimentin or MMP3 alone, or vimentin and MMP3 (Fig. 5a). The results from chamber assay indicated that miR-515-3p exerted a significant inhibitory effect on the invasion and migration of ESCC cells, whereas overexpression of vimentin or MMP3 markedly restored the invasive and migration ability of miR-515-3p-overexpressing ESCC cells (Fig. 5b and Supplementary Fig. S7a). On the contrary, the expression of vimentin and MMP3 in miR-515-3p-knockdwon ESCC cell lines (KYSE150-Luc-ZIP-miR-515-3p, EC109-ZIP-miR-515-3p) was further inhibited with shRNA (Fig. 5c). As shown in Fig. 5c, d and Supplementary Fig. S7b, knockdown of vimentin or MMP3 significantly abrogated the effects of miR-515-3p knockdown on cell invasion, migration and expressions of EMT markers in ESCC cells. Taken together, we proved that miR-515-3p suppresses cancer invasion and metastasis by directly targeting vimentin and MMP3.

### Systemic delivery of miR-515-3p suppresses tumor metastasis

**Fig. 6:**
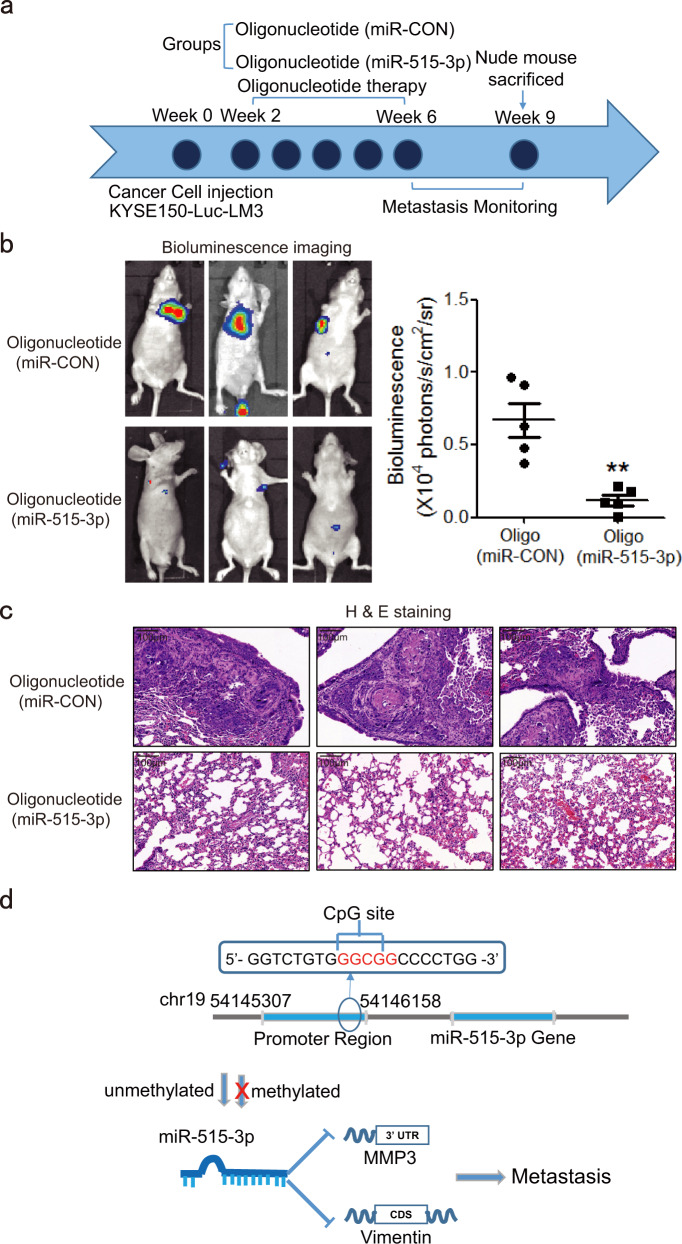
Systemic delivery of miR-515-3p suppresses tumor metastasis in vivo. **a** Diagram depicting the miR-515-3p oligonucleotide treatment experiment. **b** Lung metastasis in nude mice was detected by bioluminescence imaging and quantified. **c** H & E staining of lungs. **d** Schematic diagram of how miR-515-3p suppresses tumor metastasis by inhibiting vimentin and MMP3

Given that miR-515-3p has an essential role in ESCC metastasis, we next evaluated the therapeutic efficacy of miR-515-3p in treating metastatic ESCC cells. First, we investigated the effect of miR-515-3p oligonucleotide in ESCC cells, the cells transfected with miR-515-3p oligonucleotide had lower invasion, migration, and adhesion ability than the cells transfected with miR-CON oligonucleotide (Supplementary Fig. S8a, b). Decreased fibronectin and N-cadherin expression as well as increased E-cadherin and ZO-1 expression were observed in cells transfected miR-515-3p oligonucleotide (Supplementary Fig. S8c). Experimental metastasis model was established by intravenously injecting KYSE150-luc-LM3 cells into tail vein of nude mice, and miR-515-3p or miR-CON oligonucleotide with cholesterol modification was injected intravenously into nude mice 2 weeks later (Fig. 6a). Lung metastasis was monitored using bioluminescence imaging and confirmed by H & E staining. The results showed that systemically delivered miR-515-3p oligonucleotide significantly decreased the metastatic burden in the lungs, as compared with the group treated with miR-CON (Fig. 6b, c), suggesting that miR-515-3p may be a potential therapeutic molecule for treatment of metastatic cancer.

## Discussion

ESCC is the eighth most common malignant cancer in the world, with a 5-year survival rate of 15–25%.^21–23^ Once the cancer goes detected, patients are usually at an advanced stage. Despite many treatment strategies have been developed, metastatic spread, drug resistance, and recurrence lead to poor prognosis of the cancer patients.^24–26^ In particular, there is still no effective targeted drugs for advanced metastatic esophageal cancer. There is therefore a crying need to find prognostic biomarkers and therapeutic targets to improve the treatment outcome of advanced ESCC. Emerging evidences revealed that more than 60% of the genes in the human genome are regulated by miRNAs, which inhibit the translation of multiple target genes by binding with mRNA and thus have a key role in the physiological and pathological processes of various diseases including cancer.^27,28^ However, there is very few report regarding the identification of functional microRNAs in esophageal cancer metastasis. In our study, we identified that 6 candidate miRNAs may be involved in cancer metastasis and miR-369-3p, miR-515-3p as well as miR-331-5p were found to obviously suppress the ESCC cell invasion (Supplementary Fig. S2b). Among the three miRNAs, the miR-515-3p rarely reported in cancer became our research focus. At present, the function and upstream regulatory mechanism of miR-515-5p were reported in lung cancer and breast cancer. It has been indicated that miR-515-5p can directly bind to 3′ UTR of sphingosine kinase 1 to inhibit breast cancer cell proliferation.^29^ Moreover, in non-small cell lung cancer, miR-515-5p was found to suppress cell migration, survival and metastasis by directly targeting MARK4 and CXCL6 respectively.^30,31^ These studies suggest that miR-515-5p may an effective biomarker in cancer therapy. However, the function and underlying mechanism of miR-515-3p in cancer have not been reported. In our work, we identified abnormal expression of miR-515-3p in metastatic ESCC for the first time (Fig. 1). Attributing to its promoter hypermethylation, as indicated by whole-genome methylation sequencing and methylation-specific PCR (Fig. 2), the deregulation of miR-515-3p can be functionally correlated to the cell invasion and metastasis. As represented schematically in Fig. 6d, we here provided for the first time that miR-515-3p significantly inhibits ESCC cell invasion and metastasis through directly binding to vimentin and MMP3, rendering a significant mechanistic and functional significance in the research of miRNA in ESCC metastasis.

Metastasis is a multifaceted and systemic process that contains several essential events, dependent on the cancer cells acquiring increased cell motility, EMT and invasiveness.^32–34^ It is important to better recognize the upstream mechanisms of the key regulators of cancer metastasis. Vimentin is an important marker of EMT that has an crucial role in cancer metastasis.^35^ MMP3 can degrade extracellular matrix to promote cancer progression including invasion and metastasis.^36,37^ Here, we revealed for the first time that vimentin and MMP3 are target genes of miR-515-3p. To our knowledge, the function and action mechanism of miR-515-3p in cancer is unknown up to now. We verified that ectopic miR-515-3p expression significantly decreased the vimentin and MMP3 expression, whereas silencing of miR-515-3p increased their expression. We also verified that miR-515-3p-overexpressing cells co-overexpressing vimentin and MMP3 showed higher invasive and migration ability as compared with the miR-515-3p-overexpressing cells overexpressing vimentin or MMP3 alone, and almost completely restored the suppressive effect of miR-515-3p on ESCC invasive and migration. These data suggest that vimentin and MMP3 may not regulate each other, but have an obviously additive effect in mediating the role of miR-515-3p in invasive and metastatic potential of ESCC cells. (Fig. 5b and Supplementary Fig. S7a). We further used in vitro and in vivo assays to show that miR-515-3p directly binds to both the CDS and 3′-UTR region of vimentin and MMP3, respectively, suppressing the invasion and metastasis in ESCC (Figs. 3–5). These findings mechanistically illustrated miR-515-3p to be a novel regulator of EMT to restrain cancer metastasis, providing a useful therapeutic target for ESCC treatment.

Increasing evidences suggest that microRNA-based therapy may be an effective treatment of cancer. Currently, single-stranded miRNA inhibitors and double-stranded miRNA mimics are mainly delivered by liposomes and nanoparticle delivery.^38–40^ An in vivo experiment indicated that miRNAs can be efficiently delivered to tumor using nanoparticles embedded with hydrogel scaffold, resulting in marked reduction of breast cancer metastasis.^41^ Recent finding also indicated that use of polyethylenimine enables a systemic delivery of miRNA mimics to suppress cancer progression in vivo.^42^ In this study, given that miR-515-3p can reduce vimentin and MMP3 expression, successfully delivered miR-515-3p was expected to target vimentin and MMP3 simultaneously, which may achieve a better therapeutic effect for advanced ESCC. Our results from animal experiment confirmed that intravenous injection of miRNA-515-3p oligonucleotide significantly inhibited the metastatic spread in mice (Fig. 6), demonstrating the potential therapeutic significance of miRNA-515-3p in ESCC treatment.

This study also revealed that miR-515-3p is a useful biomarker for ESCC diagnosis and prognosis. As shown in Fig. 1, in situ hybridization analysis of miR-515-3p expression in tissue microarray suggested that downregulation of miR-515-3p in ESCC can predict lymph node metastasis and unfavorable prognosis. The expression of miR-515-3p was clearly correlated to ESCC stages and progresses, and more significantly correlated to cancer survival of ESCC (Fig. 1). These observations imply that miR-515-3p can be further explored to be a meaningful biomarker for ESCC.

In conclusion, this study demonstrates that miR-515-3p can suppress the invasive and metastatic potential of ESCC cells by directly targeting vimentin and MMP3. Promoter hypermethylation leads to the downregulation of miR-515-3p in ESCC, which correlates with unfavorable clinicopathological characteristics and poor survival of cancer patients, suggesting that miR-515-3p may be a novel diagnostic and prognostic biomarker. The preclinical data testify that systemic delivery of miR-515-3p can exert inhibitory effect on tumor metastasis, indicating the potential of developing miR-515-3p to be a new therapeutic strategy for ESCC treatment.

## Materials and methods

### Cell culture

The human ESCC cell lines KYSE150-Luc-LM3, KYSE410-Luc-I6, KYSE150-Luc,^43^ and EC109 (Chinese Academy of Sciences, Shanghai) were maintained in RPMI 1640 (Life Technologies, Gaithersburg, MD), supplemented with 10% FBS (Life Technologies) at 37 °C in 5% CO_2_ atmosphere. The human normal immortalized esophageal epithelial cell line NE1-E6E7 was kindly provided by Prof. George Tsao and Dr. Annie Cheung, School of Biomedical Sciences, The University of Hong Kong.^44^

### Boyden chamber invasion and migration assays

As previously described,^45^ cells resuspended in serum-free RPMI 1640 was seeded in the upper uncoated chamber for migration assay or upper chamber coated with matrigel (BD Biosciences, Bedford, MA) for invasion assay, and the RPMI 1640 supplemented with 20% FBS was added to the lower chamber. After 24 h, the methanol and crystal violet (0.2%) were used to fix and stain the migrated or invaded cells for 15 and 20 min, respectively, and then quantified.

### Wound healing assay

ESCC cells were spread in the 12-well plates and cultured overnight. A scratch was generated using a 10 μL pipette tip, when the cells were in a suitable density. Subsequently, cells were washed with PBS to remove damaged cells and cultured with serum-free medium for 24 h, and the wound at 0 and 24 h were photographed and then quantified with ImageJ.

### Cell viability assay

In brief, cells were spread in the 96-well plates. After 24 h, WST-1 (Beyotime Biotechnology, Shanghai, China) was added in the plates and cultured at 37 °C for 2 h. A microplate spectrophotometer (BioTek Instruments, Winooski, VT) was used to measure the absorbance (OD_450_) was measured.

### Annexin V-FITC/PI staining assay

Cells were plated in the 24-well plates, and cultured at 37 °C for 24 h. Then cells were digested with trypsin and suspended in binding buffer, subsequently stained with annexin V-FITC (KeyGen, Jiangsu, China) for 20 min and propidium iodide (PI) (KeyGen) for 10 min, respectively. Finally, cell apoptosis was analyzed.

### Cell adhesion assay

The 96-well plates were coated with matrigel (BD Biosciences), then the cells were seeded and cultured for 1 h. Next, the plates was washed with PBS, and fixed with 4% paraformaldehyde for 20 min. Subsequently, WST-1 (Beyotime Biotechnology) was added to quantify the relative adhesion ability.

### Western blotting

As previously described,^46^ the protein of cells was extract by cell lysis buffer (Cell Signaling Technology, Beverly, MA) and a certain amount of protein mixed with loading buffer was boiled at 95 °C for 10 min, subsequently electrophoresed. Next, protein was transferred to PVDF membrane (Millipore, Bedford, MA). After blocking with 5% milk for 1 h, the membrane was incubated with primary antibody for 1–2 h at room temperature. After washing three times with Tris-Buffered Saline Tween-20 (TBST), the membrane was incubated with the appropriate secondary antibody for 1 h (Cell Signaling Technology). The signal was detected using ECL (Bio-Rad, Hercules, CA) and visualized by exposure to autoradiographic film. And the antibodies used included vimentin, MMP3, ZO-1, *N*-cadherin (Cell Signaling Technology), E-cadherin, fibronectin (BD Biosciences) and Actin (Proteintech, Chicago, IL).

### RNA-sequencing

RNA was isolated by trizol (ThermoFisher Scientific, San Jose, CA), precipitated in 1:1 isopropanol (v/v) and 1 μL glycogen at −20 °C overnight. VAHTS mRNA-seq V3 Library Prep Kit was used to construct the mRNA library. Libraries were sequenced on an Illumina Hiseq X Ten sequencer for 318 cycles. Using FANSe3 algorithm, the reads longer than 17 nt, passed the Illumina quality filters were matched to the human mRNA reference database on Chi-Cloud NGS Analysis Platform (Chi-Biotech Co. Ltd., Shenzhen).

### Bioinformatics study

*miRNA-target prediction*: One software program miRanda (http://www.microrna.org/microrna/getExprForm.do) was used to predict the putative binding sites on the CDS of vimentin and 3′UTR of MMP3 for miR-515-3p.^47^

### Quantitative real-time PCR (qRT-PCR)

Trizol was used to extract the total RNA of cells and tissues, and PrimeScript II first Strand cDNA Synthesis Kit (Takara, Dalian, China) was used to perform reverse transcription, as described previously.^43,48^ SYBR Premix Ex TaqII (Takara) was used to analyze the mRNA expression of vimentin, MMP3 and internal control GAPDH on a Mini Option real-time PCR system (Bio-Rad). The primers sets: forward, 5′-TTGAACGCAAAGTGGAATC-3′, reverse, 5′-AGGTCAGGCTTGGAAACAGAGGTG-3′ for *vimentin*; forward, 5′-TGGCATTCAGTCCCTCTATGG-3′, reverse, 5′-AGGACAAAGCAGGATCACAGTT-3′ for *MMP3*; forward, 5′-GAAGGTGAAGGTCGGAGTC-3′, reverse, 5′-AAGATGGTGATGGGATTTC-3′ for *GAPDH*. The expression levels of miR-515-3p and U6 as internal control were detected with TaqMan™ MicroRNA Assay (ThermoFisher Scientific).

### Expression vector and transfection

The plasmids expressing miRNAs and the scrambled miRNA control were constructed using the BLOCK-iT^TM^ Pol II miR RNAi Expression System and Gateway technology (Invitrogen, Gaithersburg, MD), and the miRNA-knockdown plasmids were purchased from GeneCopoeia (Rockville, MD). The miR-515-3p mimic (antisense, 5′-GAGUGCCUUCUUUUGGAGCGUU-3′; sense, 5′-CGCUCCAAAAGAAGGCACUCTT-3′) and negative control as well as the miR-515-3p inhibitor (5′-AACGCUCCAAAAGAAGGCACUC-3′) and negative control were purchased from Ambion (Austin, TX). The plasmids stably expressing vimentin and MMP3, pLVX-IRES-neo-vimentin and pLVX-IRES-neo-MMP3, as well as the knockdown plasmids, were purchased from Transheep (Shanghai, China). The firefly luciferase-coding pGL3-luc plasmid (Promega, Fitchburg, WI) expressing wild-type 3′UTR of MMP3 and wild-type CDS of vimentin were generated for Luciferase reporter assay. Transfection was performed as described previously.^45^

### Site-directed mutagenesis and luciferase reporter assay

The plasmids expressing mutant CDS of vimentin and 3′UTR of MMP3 were constructed using site-directed mutagenesis kit (Agilent Technologies, Santa Clara, CA). miR-515-3p mimic or control (Ambion) were co-transfected with the wild-type or mutant CDS and 3′UTR plasmid into the ESCC cells in 24-well plates. The pRL-TK Renilla luciferase-expressing vector (Promega) was used as an internal control for normalization. After transection for 48 h, the activities of Firefly and Renilla luciferase were measured with Dual-Luciferase Reporter System (Promega) following the manufacturer’s protocol.

### DNA preparation, bisulfite DNA modification, and methylation-specific PCR

DNA of ESCC cells and tissues was isolated with HiPure Tissue DNA Mini Kit (Magen, Guangzhou, China). EZ DNA Methylation-Gold Kit™ (Zymo, Foster City, CA) was used to modify the genomic DNA following manufacturer’s protocol. For methylation-specific PCR, miR-515-3p-methylated and miR-515-3p-unmethylated primers were designed with MethPrimer.^49^ The miR-515-3p-methylated primer of CpG island (chr19: 54145907) used were: forward, 5′-TATAGAGTAAATATTTGAAATATTTTTGT-3′ and reverse, 5′-AAAAAACATTAACTACCAAAAACCGC-3′; The miR-515-3p-unmethylated primer used were: forward, 5′-TATAGAGTAAATATTTGAAATATTTTTGT-3′ and reverse, 5′-AACATTAACTACCAAAAACCACC-3′. PCR products were resolved on 2% (w/v) agarose gel for electrophoresis.

### Tissue microarray and in situ hybridization

A tissue microarray (TMA) containing 100 cases of ESCC tissues and 70 cases of paired normal tissues (Cat No: HEsoS180Su05, Shanghai Outdo Biotech, Shanghai, China) as well as a TMA containing 46 cases of normal tissues, primary tumors (T1 & 2, T3 & 4) and metastatic tumors (Cat no: HEso-Squ060CD-01, Shanghai Outdo Biotech) were used to analyze miR-515-3p expression and the correlation with clinicopathological parameters. The slide was deparaffinized in xylene and rehydrated with graded alcohol, then digested with 8 mg/mL pepsin at 3 °C for 10 min, followed by dehydration. Using microRNA ISH Buffer Set (FFPE) (Qiagen, Valencia, CA, USA, Cat No: 339450), the slide was hybridized with the probe against miR-515-3p (5′-AACGCTCCAAAAGAAGGCACTC-3′, 20 nM) (Qiagen, Cat no: 339111YD00610209-BCG) at 60 °C for 12 h. Each TMA core was scored by two pathologists who had no prior knowledge of patient data. The intensity of staining in tumor cells was categorized into four groups: score 1 (negative), score 2 (weak), score 3 (moderate), and score 4 (strong).

### In vivo experimental metastasis model and systemic miR-515-3p treatment

Female BALB/c nude mice aged 6–8 weeks were cared following the institutional guidelines and the animal experiments were approved by the Ethics Committee for Animal Experiments of Jinan University. Cells resuspended in PBS were injected intravenously into nude mice through tail vein. In the experiment involving systemic miR-515-3p treatment, miR-515-3p, or miR-CON oligonucleotide (GenePharma, Shanghai, China) was injected intravenously into mice weekly. Nine weeks after cell injection, bioluminescent imaging was performed to observe the metastasis of cancer cells (Xenogen IVIS lumima II, PerkinElmer, MA), and the signal was analyzed using Living Image R Software Version3.1. The sequences sets: antisense, 5′-CGCUCCAAAAGAAGGCACUCUU-3′, sense, 5′-GAGUGCCUUCUUUUGGAGCGUU-3′ for miR-515-3p oligonucleotide; antisense, 5′-ACGUGACACGUUCGGAGAATT-3′, sense, 5′-UUCUCCGAACGUGUCACGUTT-3′ for miR-CON oligonucleotide.

### Statistical analysis

All in vitro assays were performed three times, and all data were expressed as the mean ± SD and *t*-test was used to calculate statistically significant differences. The association between miR-515-3p expression and clinicopathological parameters was determined using Person’s rank correlation coefficient. Kaplan–Meier method was performed to analyze survival analysis with the log-rank test. *P* < 0.05 was considered statistically significant. Bars, SD; *P < 0.05; **P < 0.01; ***P < 0.001.

## Supplementary information

Supplementary Figures and Tables

## Data Availability

The data sets used and/or analyzed during the current study are available from the corresponding author on reasonable request.
