# Supplementary material for: Identification of miR-515-3p and its targets, vimentin and MMP3, as a key regulatory mechanism in esophageal cancer metastasis: functional and clinical significance
Source: Signal Transduct Target Ther. 2020 Nov 27;5:271. doi: 10.1038/s41392-020-00275-8 (PMC7693265; doi:10.1038/s41392-020-00275-8)
Supplement: Supplementary file 1 — Supplementary Figures and Tables [file 41392_2020_275_MOESM1_ESM.pdf]

Supplementary Materials for

Identification of miR-515-3p and its targets vimentin and MMP3 as a key regulatory mechanism in esophageal cancer metastasis: functional and clinical significance

Hui-Fang Hu[1], Wen Wen Xu[2], Wei-Xia Zhang[1], Xin Yan[1], Yang-Jia Li[1], Bin Li[1*], Qing-Yu He[1*]

Correspondence to: tqyhe@jnu.edu.cn； libin2015@jnu.edu.cn

This PDF file includes:

Figures. S1 to S8
Tables S1 to S2

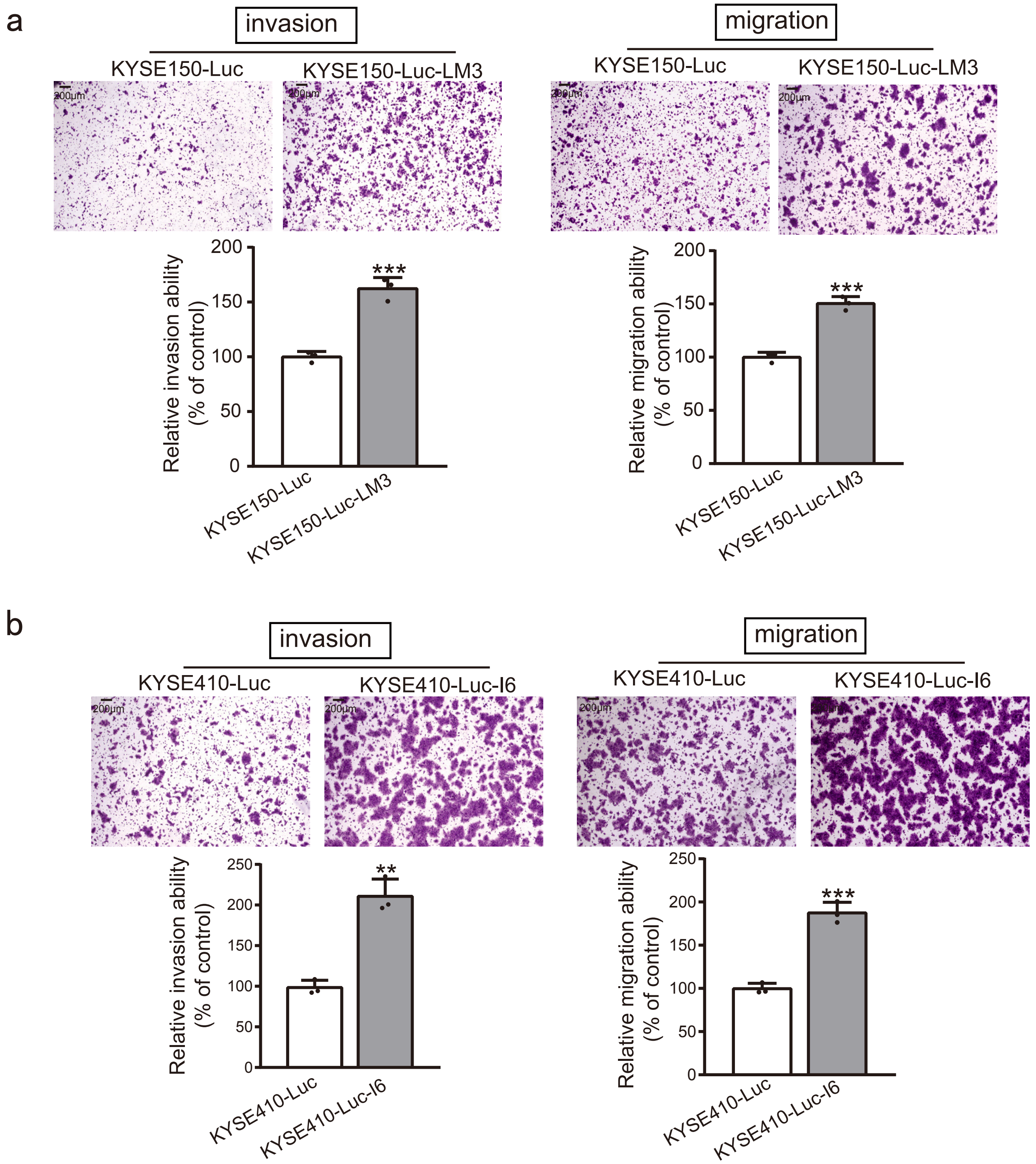

Supplementary Figure S1. Comparison of invasion and migration abilities of KYSE150-Luc-LM3 and KYSE150-Luc as well as KYSE410-Luc-I6 and KYSE410-Luc cells. (a, b) The invasion and migration abilities of KYSE150-Luc-LM3 and KYSE150-Luc cells (a) as well as KYSE410-Luc-I6 and KYSE410-Luc cells (b) were analyzed by chamber invasion and migration assays, and increased invasion and migration potential was observed in KYSE150-Luc-LM3 and KYSE410-Luc-I6 cells.

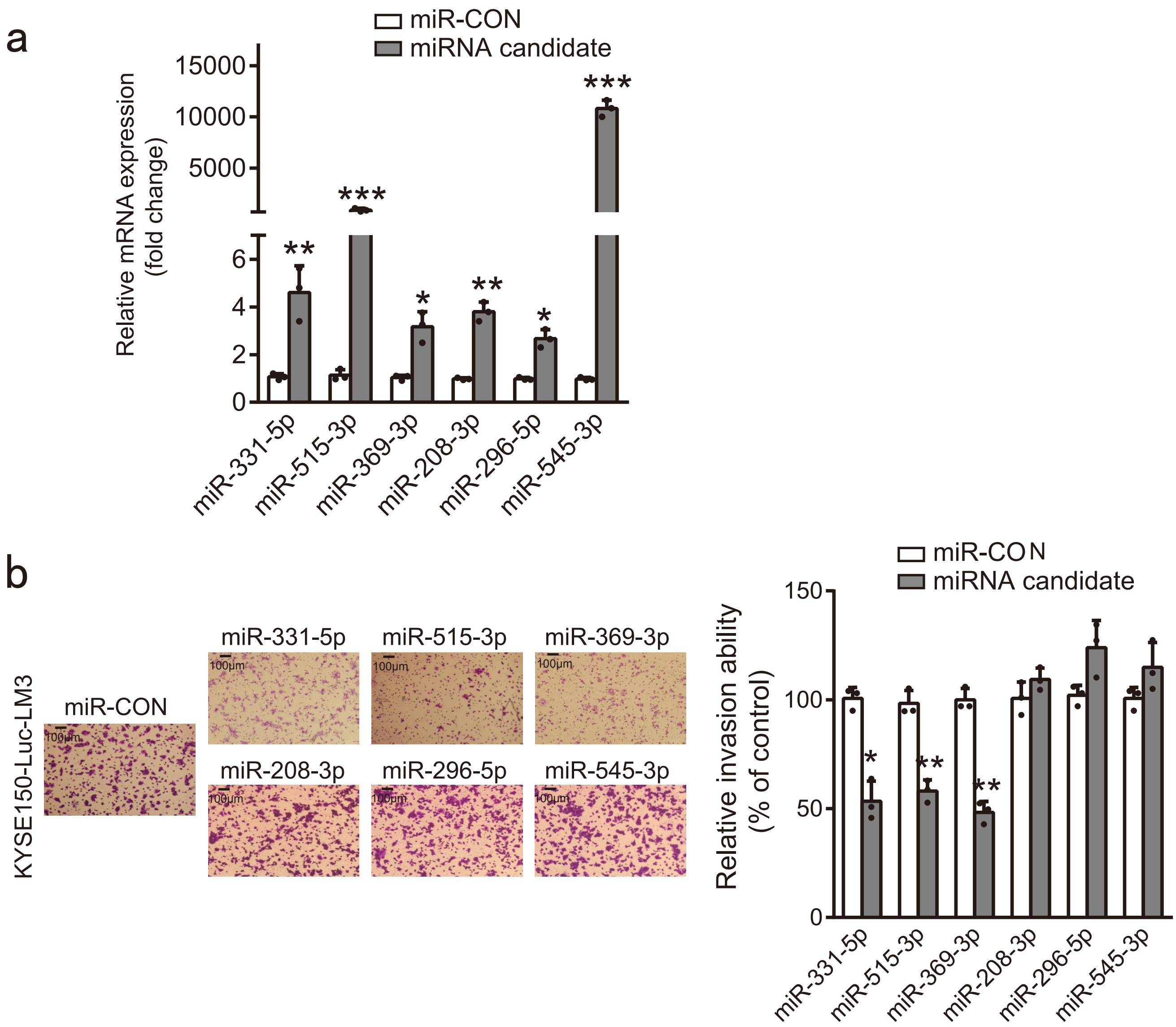

c

Survival of patients with esophageal carcinoma	
Clinical Parameter	Multivariate Log Rank *P*-value
Clinical M Status	2.31E-02
Clinical N Status	1.72E-02
Clinical Stage	2.51E-02

Supplementary Figure S2. Screening of functional miRNAs involved in cancer invasion. (a) Successful establishment of the stable cell lines overexpressing the 6 miRNA candidates indicated, respectively, in KYSE150-Luc-LM3 cells. (b) Effect of the miRNA candidates on invasive potential of ESCC cells was determined using chamber invasion assay. The quantification data indicated miR-515-3p markedly suppressed invasive ability of KYSE150-Luc-LM3 cells. (c) Correlation between miR-515-3p expression and survival of ESCC patients from OncomiR Cancer Database. Multivariate Cox analysis evaluates the combined effect of the miRNA and clinical parameters patient survival duration. A strong degree of significance indicates the likelihood that the miRNA affects survival independently of the given clinical parameter.

Supplementary Figure S2

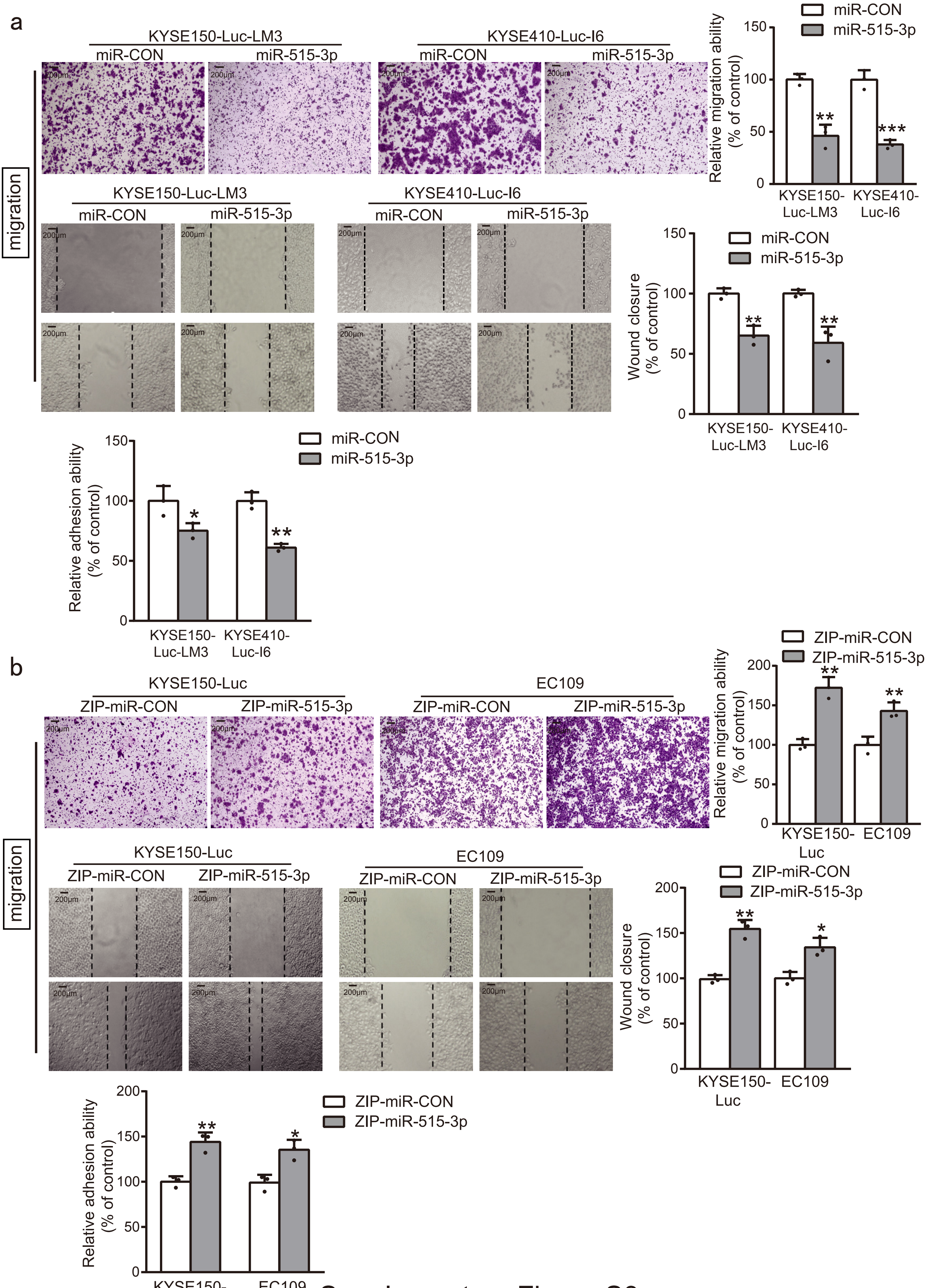

Supplementary Figure S3

Supplementary Figure S3. miR-515-3p inhibits ESCC migration and adhesion. (a) Comparison of the migration and adhesion potential of miR-515-3p-overexpressing cells and control cells by migration assay, wound healing assay and cell adhesion assay. (b) Comparison of migration and adhesion potential of miR-515-3p-knockdown cells and control cells.

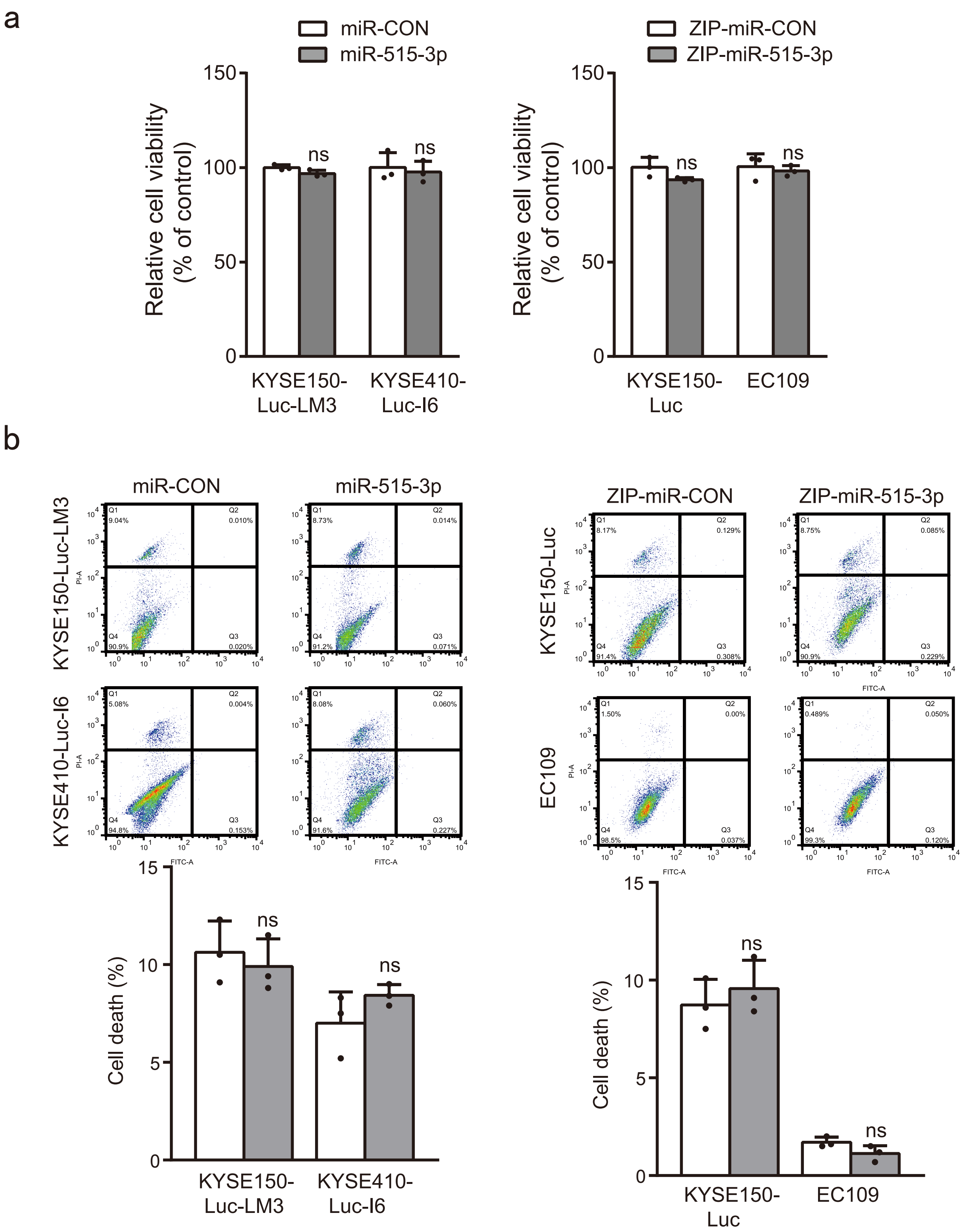

Supplementary Figure S4. miR-515-3p did not exert effect on ESCC proliferation and apoptosis. (a, b) miR-515-3p did not affect ESCC cell proliferation (a) and apoptosis (b) within 24 h, indicated by WST-1 and Annexin V-FITC/PI double staining assay.

Supplementary Figure S4

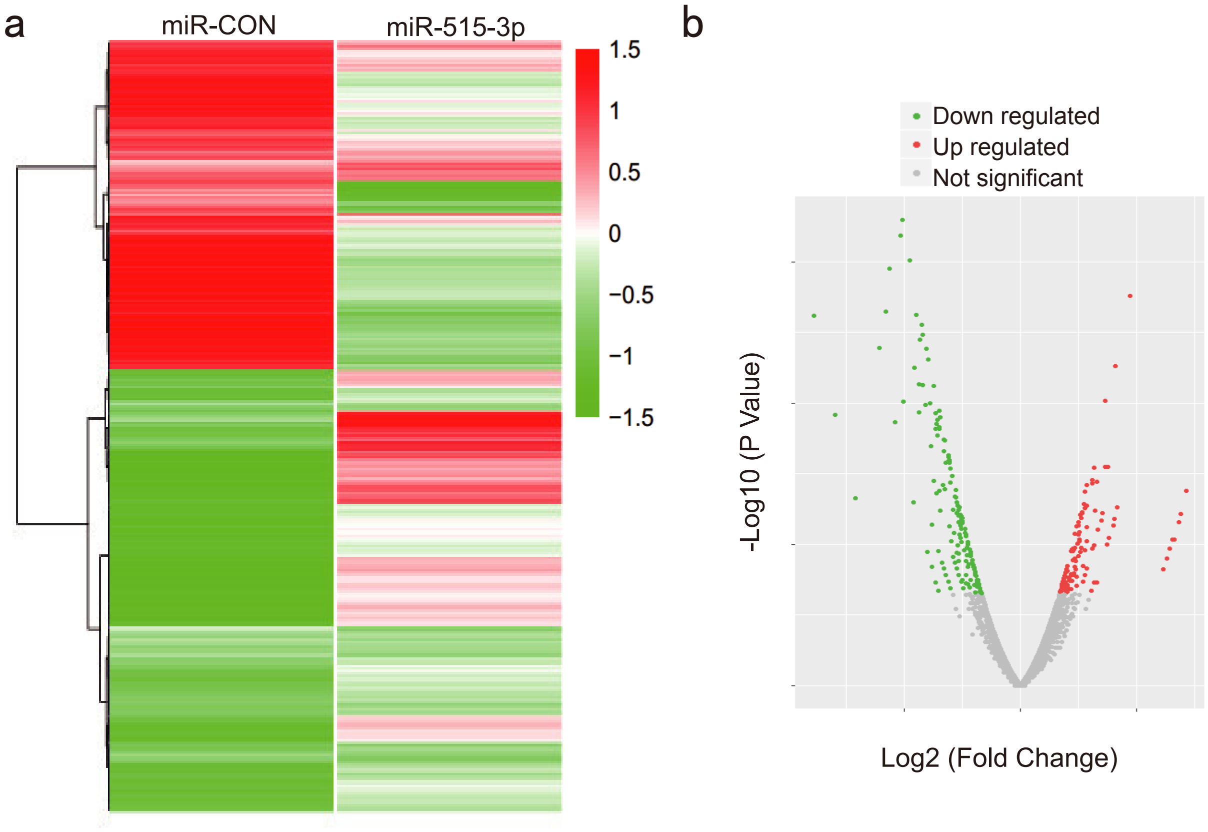

Supplementary Figure S5. Comparison of gene profiles of KYSE150-Luc-LM3-miR-515-3p and KYSE150-Luc-LM3-miR-CON cells. (a) Heatmap of the gene profiles of KYSE150-Luc-LM3-miR-515-3p and KYSE150-Luc-LM3-miR-CON cell lines analyzed by using RNA-sequencing. Red color for high abundance and green color for low abundance. (b) Volcano map of the upregulated and downregulated genes in KYSE150-Luc-LM3-miR-515-3p cells compared with KYSE150-Luc-LM3-miR-CON cells.

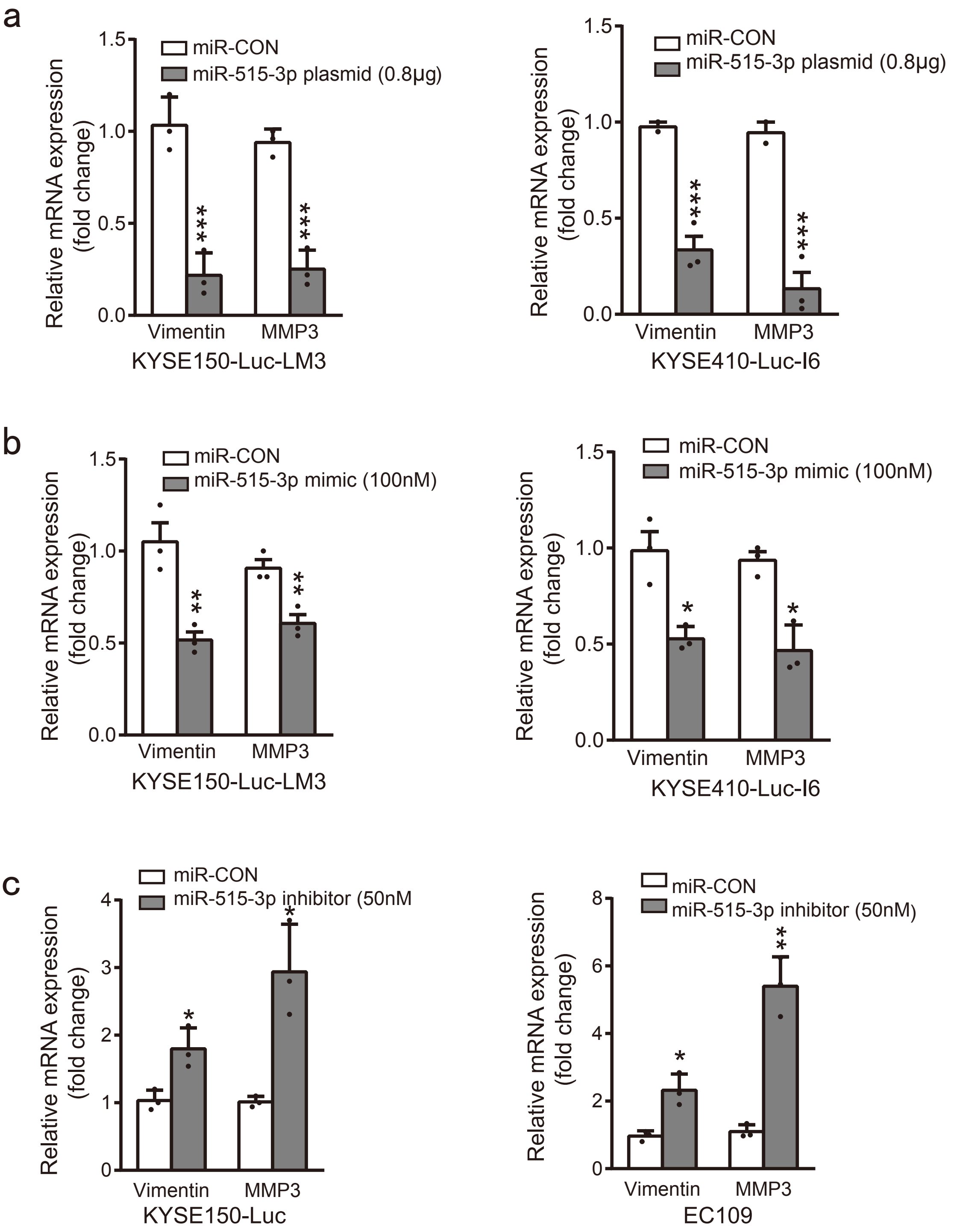

Supplementary Figure S6. Effect of miR-515-3p on vimentin and MMP3 expression at mRNA level. ESCC cells were transfected with miR-515-3p-expressing plasmid (a), miR-515-3p mimic (b), or miR-515-3p inhibitor (c), respectively, and mRNA expression of vimentin and MMP3 was compared with the control cells by qRT-PCR.

Supplementary Figure S6

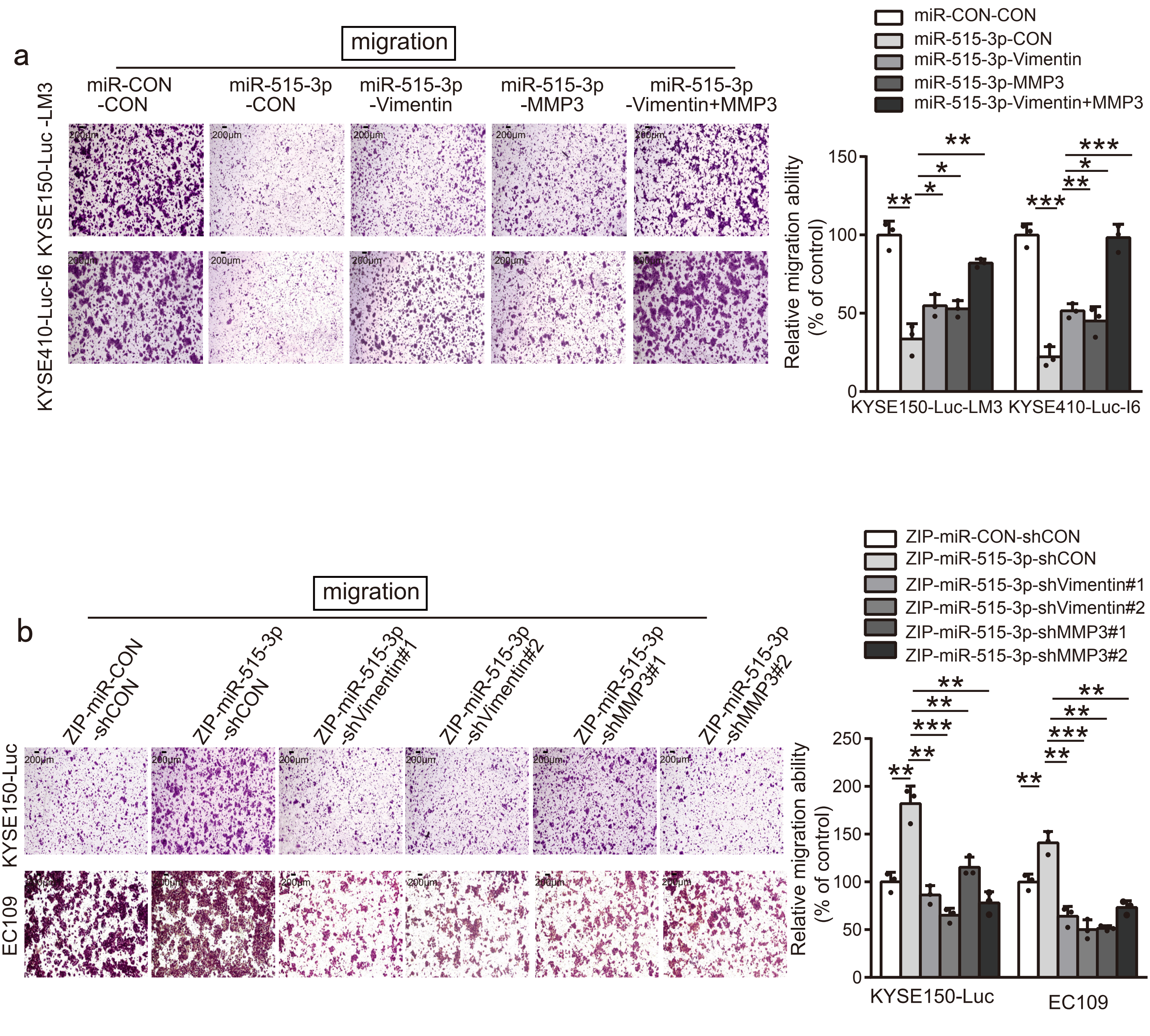

Supplementary Figure S7. Vimentin and MMP3 mediate the effect of miR-515-3p on cancer cell migration. (a) Comparison of the migration potential of the cells co-overexpressing miR-515-3p, vimentin and MMP3 or miR-515-3p and vimentin, or miR-515-3p and MMP3, as well as the control cells by chamber migration assay. (b) The migration potential of the cells were compared by chamber migration assay.

Supplementary Figure S7

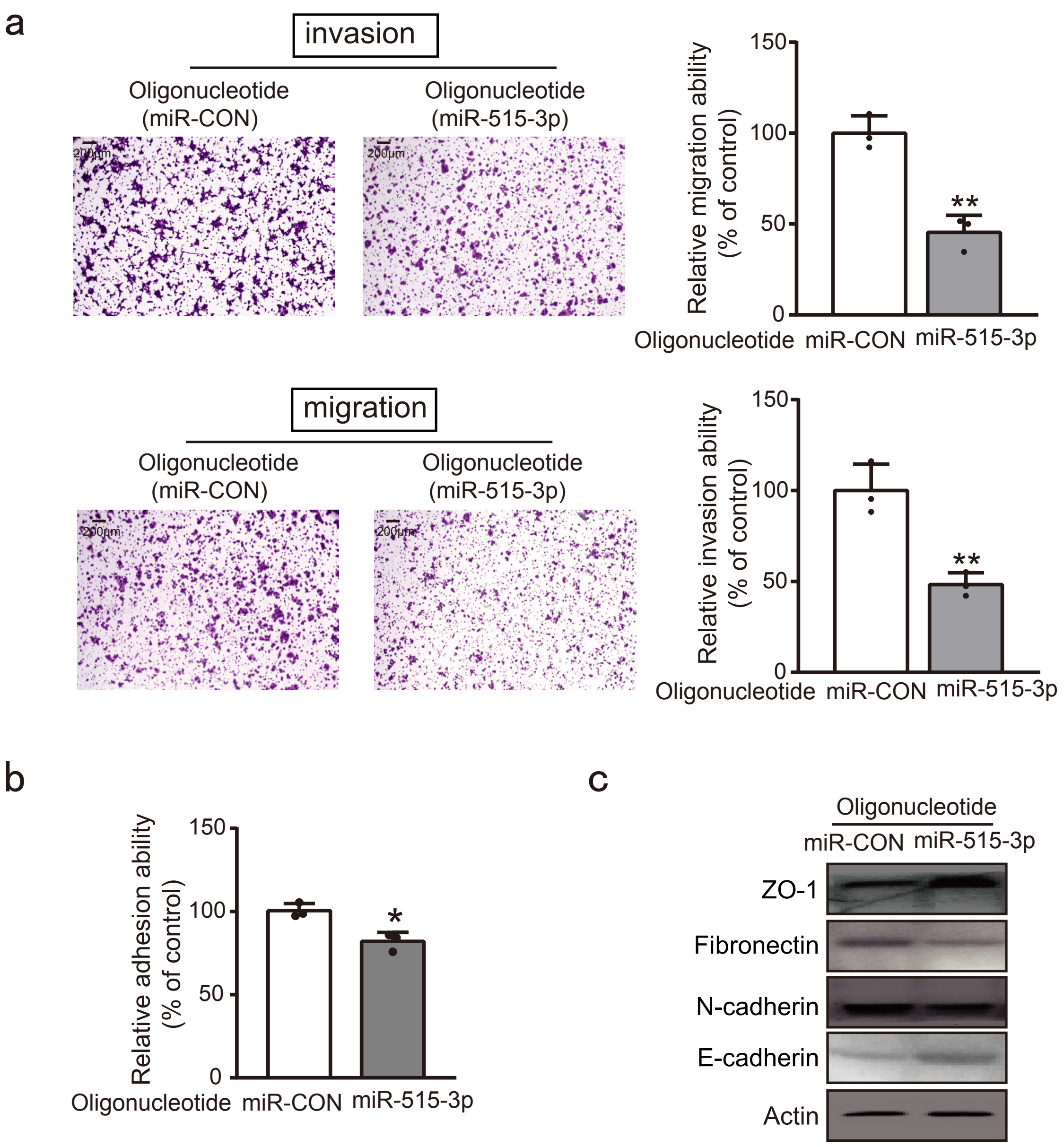

Supplementary Figure S8. miR-515-3p oligonucleotide inhibits ESCC invasion, migration and adhesion in ESCC cells. (a) migration and cell adhesion assays were performed to determine the migration and adhesion potential of cells transfected with miR-515-3p oligonucleotide and miR-CON, respectively. (b) Western blot analysis of ZO-1, fibronectin, N-cadherin and E-cadherin expression.

Supplementary Figure S8

Supplementary Table 1. Correlation between miR-515-3p expression and survival of cancer patients.

**Head and neck squamous cell carcinoma**	
**Clinical Parameter**	**Multivariate Log Rank *P*-value**
Clinical M Status	2.97E-04
Clinical N Status	1.49E-04
Clinical Stage	2.65E-04
Clinical T Status	3.36E-04
Histologic Grade	3.15E-04
Pathologic N Status	4.92E-04
Pathologic Stage	4.38E-04
Pathologic T Status	6.40E-04
**Kidney chromophobe**	
**Clinical Parameter**	**Multivariate Log Rank *P*-value**
Pathologic N Status	1.45E-04
**Kidney renal papillary cell carcinoma**	
**Clinical Parameter**	**Multivariate Log Rank *P*-value**
Pathologic M Status	0.0068
Pathologic N Status	0.0275

Multivariate Cox analysis evaluates the combined effect of the miRNA and clinical parameters patient survival duration. A strong degree of significance indicates the likelihood that the miRNA affects survival independently of the given clinical parameter.

Supplementary Table 2. Correlation between miR-515-3p expression and clinical parameters of cancer patinets.

Bladder urothelial carcinoma	
**Clinical Parameter**	**ANOVA *P*-value**
Clinical T Status	3.68E-02
Pathologic Stage	2.20E-05
Pathologic T Status	2.02E-02
**Colon adenocarcinoma**	
**Clinical Parameter**	**ANOVA *P*-value**
Pathologic M Status	4.43E-02
**Kidney chromophobe**	
**Clinical Parameter**	**ANOVA *P*-value**
Pathologic M Status	7.64E-07
Pathologic Stage	2.67E-04
Pathologic T Status	2.84E-07
**Kidney renal clear cell carcinoma**	
**Clinical Parameter**	**ANOVA *P*-value**
Pathologic T Status	2.51E-10
**Kidney renal papillary cell carcinoma**	
**Clinical Parameter**	**ANOVA *P*-value**
Pathologic N Status	4.45E-02
Pathologic T Status	4.47E-03
**Lung adenocarcinoma**	
**Clinical Parameter**	**ANOVA *P*-value**
Pathologic M Status	6.79E-04
Pathologic Stage	3.65E-03
**Skin cutaneous melanoma**	
**Clinical Parameter**	**ANOVA *P*-value**
Pathologic N Status	1.87E-02
**Testicular germ cell tumors**	
**Clinical Parameter**	**ANOVA *P*-value**
Clinical T Status	1.11E-03
Pathologic T Status	2.96E-02
**Uterine corpus endometrial carcinoma**	
**Clinical Parameter**	**ANOVA *P*-value**
Clinical Stage	3.69E-04
**Uveal melanoma**	
**Clinical Parameter**	**ANOVA *P*-value**
Clinical M Status	2.51E-05
Clinical N Status	3.88E-06

Analysis of variance (ANOVA) compares miRNA expression between the different cohorts within each clinical parameter.
